# The boundaries of our imagination are not restricted by limits, but by lack of knowledge

**DOI:** 10.1186/s13049-019-0677-4

**Published:** 2019-10-30

**Authors:** Kristi G. Bache, Marius Rehn

**Affiliations:** 1 Department of Research, The Norwegian Air Ambulance Foundation, Oslo, Norway; 2 University of Oslo, Institute of Basic Medical Science, Oslo, Norway; 3 Pre-hospital Division, Air Ambulance Department, Oslo University Hospital, Oslo, Norway; 4 Faculty of Health Sciences, University of Stavanger, Stavanger, Norway

In a world with increased globalization, geographically distant populations are often exposed to similar challenges and hazards; the solutions to these problems differ dependent on environment and resource. In our Western part of the world, the summer season that delivers longed-for good weather also brings a terrible toll by claiming lives to drowning. Although modest in numbers, just as devastating to family and friends left behind, and the message goes out to reinforce the crucial task of bystanders in providing basic lifesaving support. Because it takes time for professional help to arrive, and the help that makes the ultimate difference is often found exclusively in hospitals. And herein lays a classic controversy: Should rapid transport to advanced care in the hospital overrule initiating critical care at the scene? Is it even feasible with critical care on site, or are we wasting time? However, this is a false controversy. When limited time is all we have, the question is not which is better, but rather how we can improve quality of care at every stage because strengthened prehospital care is the only alternative to improve survival.

The impact of a physician-manned Helicopter Emergency Medical Service (HEMS) is hard to quantify [1], and reasons for this includes lack of use of sound methodology, population heterogeneity, and challenges related to outcome measures and data collection under critical conditions [2]. Helicopters are resource-intensive and are associated with the fear of draining limited funds from the in- to pre-hospital services. This concern has resulted in a great impetus to demonstrate its independent effects, as if prehospital and in-hospital care were competing with each other and not caring for the same critically ill patient. We believe a shift in awareness from our own environment to a distant population might illustrate our point.

In the small country of Bhutan, with mountains raising from just under a hundred meters above the sea level to as high as 7500 m into the sky, and a population of about 1 million people scattered in towns and villages throughout the eastern Himalaya, the logistics related to healthcare and emergent retrieval and treatment of acute illness or trauma are very challenging. To address these geographical challenges, the government of Bhutan provides healthcare for free to all of its citizens and has established a network of ‘basic health units’ in villages throughout the country. Further referral to a district hospital ensures medical help provided by a physician, although intensive care, emergency medicine and surgery is only provided for by one of the three regional hospitals in the country. Most of the population is left with poor access to emergency and critical care. This situation was not acceptable to the Kingdom of Bhutan, which emphasizes on wisdom, power and compassion in the governing of the country, and embedded the measure GNH (gross national happiness) into their constitution [3] instead of the familiar economy- related GNP (gross national product). Fueled by the concept of delivering a competent physician and nurse to those in critical need and with insight from existing services in other countries, the idea of a critical care helicopter retrieval team for Bhutan was born. Nine months later the first rescue flight was a fact; inspired by the futile death of a child, aiming to rescue the many more in sight.

Two years later, we proudly present the first international report from Bhutan HEMS and, to our knowledge, the first published report on the successful pre-hospital administration of surfactant. In a modest way, and in a form low in rung of the hierarchy of study design, this case report envisions our above mentioned point: the impact of a physician and a helicopter as documented by the gold standard for measuring effect, an RCT which sits at the top of the hierarchy, is challenging for many reasons and might in fact prove unethical (i.e. how would you randomize?). But this neonate would not have survived without pre-hospital critical care. Advanced medical interventions are insignificant if beyond the reach of the patient in need. Our testimony remains: there is no alternative but to strengthen pre-hospital critical care.
